# Real-world effectiveness of erenumab in Japanese patients with migraine

**DOI:** 10.1016/j.heliyon.2024.e26568

**Published:** 2024-02-17

**Authors:** Keisuke Suzuki, Shiho Suzuki, Tomohiko Shiina, Yasuo Haruyama, Saro Kobayashi, Mukuto Shioda, Koichi Hirata

**Affiliations:** a Department of Neurology, Dokkyo Medical University, Japan; b Integrated Research Faculty for Advanced Medical Sciences, Dokkyo Medical University, Japan

**Keywords:** Migraine, Erenumab, Calcitonin gene-related peptide

## Abstract

**Background:**

Real-world evidence of erenumab effectiveness in migraine patients in Asia with various comorbidities and multiple previous medication failures is still limited.

**Methods:**

A 6-month single-center cohort study of 45 patients with episodic or chronic migraine (CM) treated with erenumab was conducted. In the cohort, 60.0% were switching from other calcitonin gene-related peptide monoclonal antibodies (CGRP mAbs), and 66.7% had ≥4 prophylaxis failures. The change in monthly migraine days (MMDs) from baseline and percentages of responders after treatment were calculated. Weekly migraine days (WMDs) were obtained at baseline and at months 1, 2 and 3 and were compared between weeks 2 and 4.

**Results:**

In total, 36%, 47%, and 63% of patients had a ≥30% response at 1, 3, and 6 months, respectively. The cumulative percentage of patients achieving a ≥30% response over 6 months was 85%. Early responders (average ≥ 30% response at 1–3 months) accounted for 37.8%, 55.6%, and 25.9% of the total, CGRP mAb-naïve, and CGRP mAb-switching groups, respectively. Late responders (average < 30% response at 1–3 months and average ≥ 30% response at 4–6 months) accounted for 46.4%, 37.5%, and 58.8% of nonearly responders in the total, CGRP mAb-naïve, and CGRP mAb-switching groups, respectively. Mild adverse reactions were observed in 5 patients (11.1%). Wearing-off, defined as an increase in the number of WMDs ≥2 between week 2 and week 4, was observed in 2.4–12.5% at months 1–3.

**Conclusion:**

Erenumab was effective in migraine patients. At least 4–6 months may be preferable for efficacy evaluation in patients switching to erenumab from other CGRP mAbs.

## 1 Introduction

Migraine is a highly disabling neurological disorder, exhibiting an annual prevalence rate of 15% for active cases, peaking in the 30 s [1]. Migraine is characterized by pulsatile, moderate to severe headache, with accompanying symptoms such as photophobia, phonophobia and nausea occurring during the prodromal and headache phases. Patients with migraine report reduced work productivity, loss of carrier potential and impact on children's academic performance and school participation; furthermore, the burden during an interictal period, a period between migraine attacks or a nonheadache phase of an attack, and the cost of the disease are also important social issues [2]. Prophylactic treatment is needed for migraine patients who have headaches for four or more days a month and with at least some disability [3]. However, conventional migraine prophylactic drugs such as beta-blockers, antiepileptic drugs, and antidepressants were developed for clinical conditions other than migraine and have limitations in efficacy and tolerability. Therefore, the development of new prophylactic medications with high efficacy and safety related to the pathophysiology of migraine has long been awaited.

Calcitonin gene-related peptide (CGRP), widely expressed in both the peripheral and central nervous systems, has been found to play a major role in the pathophysiology of migraine through dysfunctional activation of the trigeminovascular nociceptive system. There are two therapeutic classes of agents that inhibit CGRP signaling: monoclonal antibodies (mAbs) and small molecule antagonists (gepants) [4]. Currently, there are four CGRP mAbs targeting CGRP (galcanezumab, fremanezumab and eptinezumab) or its receptor (erenumab) [5]. The use of mAbs acting on CGRP or its receptor was previously recommended for migraine patients for whom at least two of the available prophylactic treatments had failed [6,7], but the updated European Headache Federation guidelines have positioned CGRP mAbs as the first-line treatment option for migraine prophylaxis [8]. Erenumab is the only fully human mAb that binds to the CGRP receptor approved for migraine prophylaxis. The efficacy and safety profiles of erenumab have already been established in clinical trials [[9], [10], [11]] and real-world studies [[12], [13], [14], [15]]. The latter clinical data show the efficacy of erenumab on chronic migraine that fails to respond to ≥2 [14] or ≥4 migraine preventive medication classes [15] and medication overuse headache (MOH) [13]. However, clinical evidence of erenumab use in patients with migraine with various comorbidities and multiple previous medication failures in Asia, including Japan, is still limited.

In this study, we aimed to evaluate the effectiveness of erenumab in patients with migraine, including patients with chronic migraine (CM), patients with MOH, patients switching from other CGRP mAbs, and patients with difficult-to-treat migraine (DTT), in a single center in Japan. Our observational cohort study including patients with refractory migraine may provide useful clinical evidence for the efficacy and safety of erenumab treatment in Asia.

## 2 Methods

### 2.1 Study design

This retrospective, single-center cohort study with a 6-month observation period was designed to examine the efficacy and safety of erenumab in migraine patients. The patients included in this study were derived from the erenumab group of our observational study of 228 patients who received the three different types of CGRP mAbs [16] with additional follow-up. This study was approved by the Institutional Review Board of Dokkyo Medical University. All participating patients were informed about the study and had the opportunity to opt out of participating in the study. Our Institutional Review Board waived the requirement for patients to provide written informed consent given the retrospective, observational nature of this study.

## 3 Patients

1) Inclusion criteria

Among episodic migraine (EM) or CM patients aged 18 years or older who attended our headache outpatient clinic and received erenumab from October 2022 to January 2023, 45 patients with migraine with at least 3 months of follow-up were included in the study. Patients had received at least two months of treatment with one or more prophylactic agents, including other types of CGRP mAbs, prior to starting erenumab treatment. Patients consistently received 70 mg erenumab. In Japan, only 70 mg of erenumab monthly was approved, and the dose could not be increased to 140 mg. The inclusion criteria for this study were adults with a confirmed diagnosis of migraine and at least 3 months of follow-up after treatment with erenumab.

2) Exclusion criteria

Exclusion criteria were inadequate documentation in headache diaries, age less than 18 years, and the presence of organic brain lesions that could cause headache.

## 4 Diagnosis of migraine

Migraine with aura and migraine without aura were diagnosed by a headache specialist according to the International Classification of Headache, 3rd edition (ICHD-3) [17]. CM was defined as headache lasting at least 15 days per month for at least 3 months, during which migraine features were present at least 8 days per month; EM was defined as headache occurring 4–14 days per month. MOH was diagnosed according to the ICHD-3 [17]. Patients with DTT migraine were defined as those with MOH or psychiatric comorbidities.

## 5 Clinical evaluation

The number of monthly migraine days (MMDs) before and 1–6 months after erenumab administration were obtained from headache diaries, and changes in the number of MMDs from baseline were calculated. Data on the duration of migraine, comorbidities, prior prophylactic medications, aura and accompanying symptoms (nausea, photophobia, phonophobia, osmophobia and allodynia), body mass index, and migraine characteristics were obtained from clinical records. Adverse reactions reported in clinical records were investigated. We defined the CGRP mAb-naïve group as those who had never used CGRP mAb and CGRP mAb-switching groups as those who had used CGRP mAb but switched to erenumab from other CGRP mAbs due to insufficient response or adverse reactions. The percentage decrease in the number of MMDs from baseline was calculated, and the response rates at 1–6 months were grouped as <30%, 30–49%, 50–74%, and 75–100%. Based on the percentage of MMD reduction after erenumab treatment, we defined the group with an MMD reduction of ≥30% as responders and the group with an MMD reduction of <30% as nonresponders. Early responders were defined as patients with an average response rate of ≥30% at 1–3 months, and late responders were defined as patients with an average response rate of <30% at 1–3 months and ≥30% at 4–6 months. Clinical factors associated with an average percentage reduction in the number of MMDs of ≥30% over 6 months were evaluated. The cumulative percentage of patients achieving a ≥30% response at 1–6 months was also analyzed. To assess the conversion rate from CM to EM at baseline and 1–6 months, patients were classified as CM (15≥MMDs), high-frequency EM (HFEM, 8≤MMDs≤14), medium-frequency EM (MFEM, 4≤MMDs≤7), or low-frequency EM (LFEM, 3≤MMDs) [18].

The number of weekly migraine days (WMDs) was obtained at baseline and at months 1, 2 and 3. The number of WMDs at baseline was calculated by dividing the baseline number of MMDs by 30 and multiplying by 7 [19]. The MMD change, response rate, and early and late responder rates were evaluated in the total (n = 45), CGRP mAb naïve (n = 18), and CGRP mAb-switching groups (n = 27). A “wearing-off effect” was defined as a ≥2-day increase in WMDs from week 2 to week 4 [20].

### 5.1 Statistical analysis

Sample size calculation was not performed given the real-world setting of this study. To compare nonresponders and responders, the Mann‒Whitney *U* test or Student's *t*-test was used for the continuous variables (age, disease duration, body mass index and baseline number of MMDs) after confirming the normal or nonnormal distribution by the Shapiro‒Wilk test. The Chi-square test, Fisher's exact test or Fisher-Freeman-Halton exact test was used for the categorical variables (presence of aura, CM, MOH, DTT migraine, daily headache, pain location, pain characteristics, accompanying symptoms, prior prophylaxis failures, switching from other CGRP mAbs, and comorbidities). A generalized linear mixed effect model (GLMM) followed by a Bonferroni post hoc correction was used to determine a significant difference in MMD changes from baseline to each month after erenumab treatment in the total cohort, the CGRP mAb-naïve group and the group of patients that switched from other CGRP mAbs to erenumab. The mean changes in WMDs were compared using the Wilcoxon signed rank test between W2 and W4 in months 1, 2, and 3. The sensitivity analysis including 41 patients who completed 6 months of treatment was performed using a GLMM followed by Bonferroni post hoc correction.

Two-tailed *p* < 0.05 was considered statistically significant. IBM SPSS Statistics version 28 (IBM SPSS, Tokyo, Japan) was used for all statistical analyses. GraphPad Prism for Mac (Version 8; GraphPad Software, San Diego, USA) and Microsoft Excel version 16.18 were used to create figures.

## 6 Results

A total of 4 patients (8.9%) discontinued treatment after receiving erenumab due to an inadequate response at month 4. Table 1 shows the baseline characteristics of the patients. In the total cohort, the percentages of patients with CM, MOH, DTT migraine, daily headache, and switching from other CGRP mAbs were 77.8%, 55.6%, 57.8%, 15.6%, and 60%, respectively. The baseline number of MMDs was 20.1 ± 6.4.

**Table 1:** Baseline patient characteristics.

	Total
n (M/F)	45 (3/42)
Age, mean ± SD, years	44.1 ± 13.3
Disease duration, mean ± SD, years	24.7 ± 12.5
Body mass index, mean ± SD, kg/m^2^	22.4 ± 3.1
Migraine with aura, n (%)	4 (8.9)
CM, n (%)	35 (77.8)
MOH, n (%)	25 (55.6)
DTT migraine, n (%)	26 (57.8)
Daily headache, n (%)	7 (15.6)
Pain location, n (%)	
Unilateral	17 (37.8)
Bilateral	33 (73.3)
Pain characteristics, n (%)	
Pulsating	33 (73.3)
Pressing	29 (64.4)
Accompanying symptoms, n (%)	
Nausea	36 (80.0)
Photophobia	42 (93.3)
Phonophobia	38 (84.4)
Osmophobia	20 (44.4)
Allodynia	14 (31.1)
Prior prophylaxis failures, n (%)	
1	2 (4.4)
2	2 (4.4)
3	11 (24.4)
≥4	30 (66.7)
Switching from other CGRP mAbs, n (%)	27 (60.0)
Baseline number of MMDs, mean ± SD	20.1 ± 6.4
Comorbidities, n (%)	24 (53.3)
Psychiatric disorders, n (%)	7 (15.6)

CGRP mAb = calcitonin gene-related peptide monoclonal antibody; CM = chronic migraine; DTT migraine = difficult-to-treat migraine; MMDs = monthly migraine days; MOH = medication overuse headache.

**Fig. 1:**
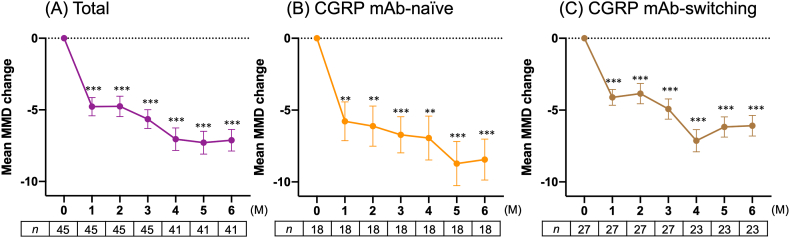
Mean changes in the number of monthly migraine days from baseline after erenumab treatment. (A) Total, (B) CGRP mAb-naïve and (C) CGRP mAb-switching groups. **p < 0.01, ***p < 0.001, compared to baseline; a generalized linear mixed-effects model followed by a Bonferroni post hoc correction was used.

After erenumab treatment, the number of MMDs significantly decreased from baseline in the total, CGRP mAb-naïve and CGRP mAb-switching groups (Fig. 1). In total, the MMD reduction was −4.8 ± 4.3, −5.6 ± 4.4, and −7.1 ± 4.8 days at 1, 3, and 6 months, respectively. The number of MMDs decreased from baseline by −5.8 ± 5.7, −6.7 ± 5.3, and −8.4 ± 6.0 days at 1, 3, and 6 months in the CGRP mAb-naïve group, respectively, and by −4.1 ± 2.9, −4.9 ± 3.6, and −6.1 ± 3.4 days from baseline in the group of patients that switched from other CGRP mAbs, respectively.

**Fig. 2:**
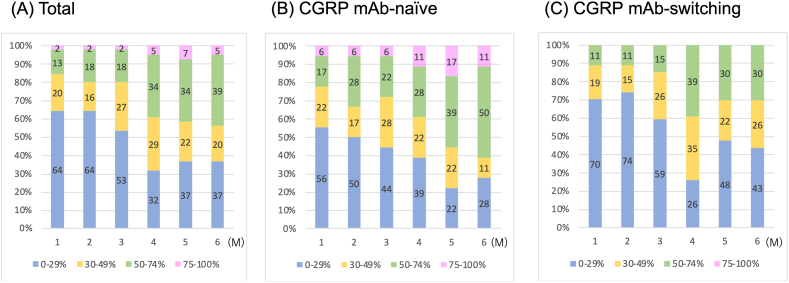
Proportions of responders after erenumab treatment. (A) Total, (B) CGRP mAb-naïve and (C) CGRP mAb-switching groups.

The total and CGRP mAb-naïve groups had favorable response rates at 1–6 months relative to the CGRP mAb-switching group. The percentages of responders after erenumab treatment at 1–6 months are shown in Fig. 2. In total, the percentages of patients with a ≥30% response at 1, 3, and 6 months were 36%, 47%, and 63%, respectively. In the CGRP mAb-naïve group, the percentages of patients with a ≥30% response rate at 1, 3, and 6 months were 45%, 56% and 72%, respectively. In the CGRP mAb-switching group, the percentages of patients with a ≥30% response rate at 1, 3, and 6 months were 30%, 41%, and 56%, respectively.

In the analysis including 41 patients who completed the 6-month follow-up, significant mean MMD reductions from baseline to each time point over 6 months (−5.2 ± 4.3 at 1 month, −6.0 ± 4.4 at 3 months, and −7.1 ± 4.8 days at 6 months) were comparable to the entire cohort results (Supplementary Fig. 1). The percentages of ≥30% responders at 1, 3, and 6 months were 39%, 49%, and 63%, respectively (Supplementary Fig. 2).

**Fig. 3:**
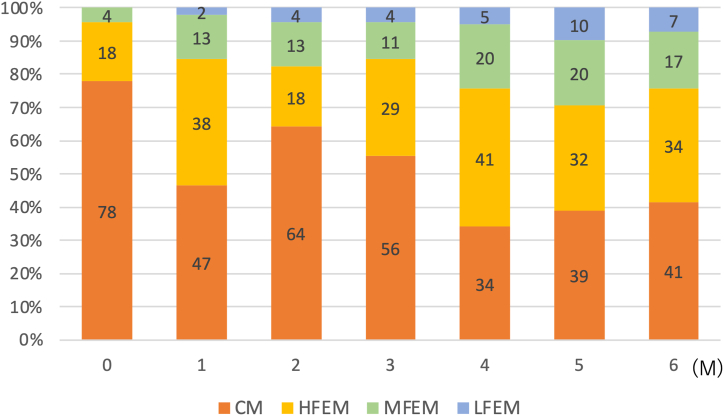
Proportions of patients with CM, HFEM, MFEM and LFEM from baseline to 1–6 months. CM = chronic migraine (15≥MMDs); HFEM = high-frequency episodic migraine (8≤MMDs≤14); MFEM = medium-frequency episodic migraine (4≤MMDs≤7); LFEM = low-frequency episodic migraine (3≤MMDs).

**Fig. 4:**
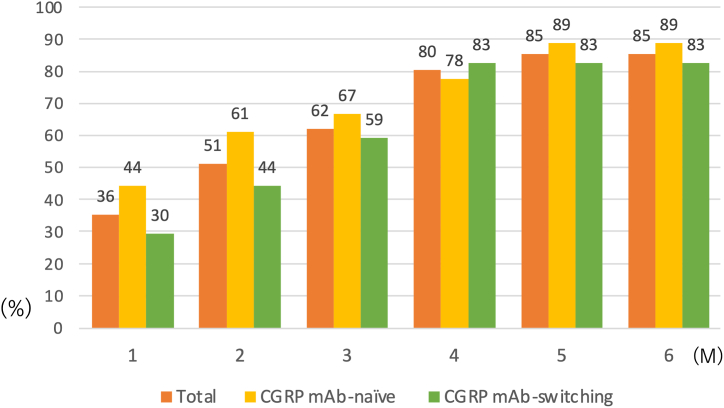
Cumulative percentage of patients achieving a ≥30% response.

**Fig. 5:**
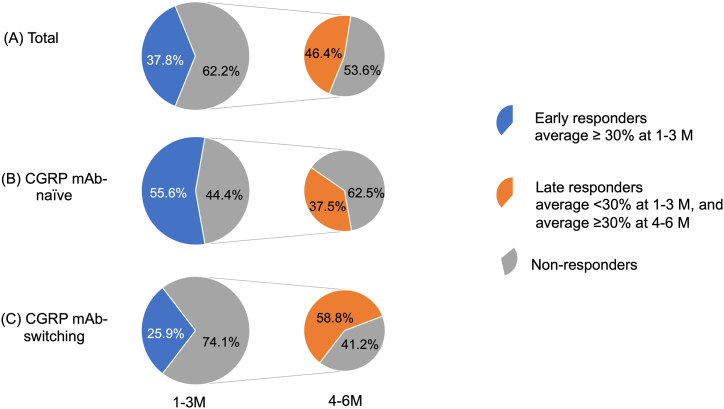
Proportions of early and late responders.

**Fig. 6:**
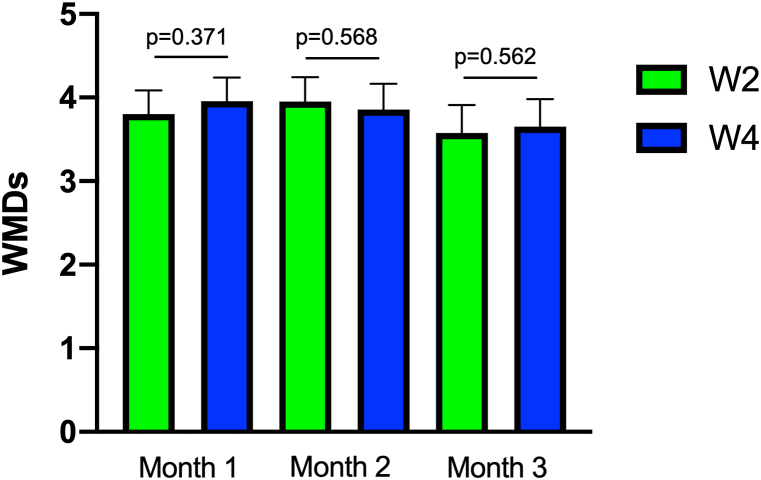
Mean changes in the number of weekly migraine days at months 1, 2, and 3. The Wilcoxon matched-pairs signed rank test was used.

Fig. 3 shows the changes from baseline to 1–6 months in the percentage of patients with CM, HFEM, MFEM, and LFEM. The percentage of patients with CM was 78% at baseline, which decreased to 56% at 3 months and 41% at 6 months. The percentage of patients with HFEM was 18% at baseline, which increased to 29% at 3 months and 34% at 6 months. The percentage of patients with LFEM increased from 0% at baseline to 4% at 3 months and 7% at 6 months. Fig. 4 illustrates the cumulative percentage of patients achieving a ≥30% response. The percentages increased over time, with the cumulative percentages of patients who achieved a ≥30% response over 6 months being 85%, 89%, and 83% in the total, CGRP mAb-naïve, and CGRP mAb-switching groups, respectively. The percentages of patients who were early responders (average ≥ 30% response at 1–3 months) were 37.8% (17/45) in the total, 55.6% (10/18) in the CGRP mAb-naïve group, and 25.9% (7/20) in the CGRP mAb-switching group. The percentages of patients who were late responders (average < 30% response at 1–3 months and average ≥ 30% response at 4–6 months) were 46.4% (13/28), 37.5% (3/8), and 58.8% (10/17) of nonearly responders in the total, CGRP mAb-naïve, and CGRP mAb-switching groups, respectively (Fig. 5). When looking at the change in the number of migraine days per week, we found that the numbers of WMDs were not significantly different between week 2 and week 4 at 1, 2, and 3 months (Fig. 6). A wearing-off effect, defined as a ≥2-day increase in WMDs from week 2 to week 4, was observed in 2 (4.4%), 1 (2.4%), and 5 (12.5%) patients at months 1, 2, and 3, respectively. Table 2 shows a comparison of the clinical factors for ≥30% responders and nonresponders at months 1–6. Responders were associated with a lower rate of CM, a lower number of baseline MMDs and a greater rate of photophobia.

**Table 2:** Clinical factors related to an average ≥ 30% reduction in the number of MMDs over 6 months.

	Nonresponders	Responders	*p* value
n (M/F)	20 (1/19)	25 (2/23)	1.000
Age, mean ± SD, years	43.6 ± 11.4	44.6 ± 14.9	0.804
Disease duration, mean ± SD, years	25.9 ± 12.1	23.8 ± 13.0	0.591
Body mass index, mean ± SD, kg/m^2^	22.8 ± 3.1	22.1 ± 3.1	0.410
Migraine with aura, n (%)	0 (0.0)	4 (16.0)	0.117
CM, n (%)	19 (95.0)	16 (64.0)	0.013
MOH, n (%)	12 (60.0)	13 (52.0)	0.592
DTT migraine, n (%)	13 (65.0)	13 (52.0)	0.380
Daily headache, n (%)	4 (20.0)	3 (12.0)	0.682
Pain location, n (%)			
Unilateral	7 (35.0)	10 (40.0)	0.731
Bilateral	16 (80.0)	17 (68.0)	0.366
Pain characteristics, n (%)			
Pulsating	15 (75.0)	18 (72.0)	0.821
Pressing	15 (75.0)	14 (56.0)	0.186
Accompanying symptoms, n (%)			
Nausea	17 (85.0)	19 (76.0)	0.453
Photophobia	17 (85.0)	25 (100.0)	0.045
Phonophobia	18 (90.0)	20 (80.0)	0.358
Osmophobia	10 (50.0)	10 (40.0)	0.502
Allodynia	8 (40.0)	6 (24.0)	0.249
Prior prophylaxis failures, n (%)			0.665
1	0 (0.0)	2 (8.0)	
2	1 (5.0)	1 (4.0)	
3	4 (20.0)	7 (28.0)	
≥4	15 (75.0)	15 (60.0)	
Switching from other CGRP mAbs, n (%)	13 (65.0)	14 (56.0)	0.540
Baseline number of MMDs, mean ± SD	23.0 ± 4.7	17.8 ± 6.8	0.006
Comorbidities, n (%)	11 (55.0)	13 (52.0)	0.841
Number of comorbidities, n (%)			0.744
0	11 (55.0)	12 (48.0)	
1	7 (35.0)	8 (32.0)	
2	2 (10.0)	4 (16.0)	
3	0 (0.0)	1 (4.0)	

CGRP mAb = calcitonin gene-related peptide monoclonal antibody; CM = chronic migraine; DTT migraine = difficult-to-treat migraine; MMDs = monthly migraine days; MOH = medication overuse headache.

The *p* value was calculated with Student's *t*-test for continuous variables and with the chi-squared test, Fisher's exact test or Fisher-Freeman-Halton exact test for categorical variables.

Adverse reactions were observed in 5 patients (11.1%); 1 patient (2.2%) had an injection site reaction, and 4 patients (8.9%) had constipation. None of these patients needed treatment, and all had mild symptoms.

## 7 Discussion

This study evaluated the efficacy and safety of erenumab in patients with refractory migraine. In the cohort, 78% had CM, 56% had MOH and 60% were switching from other CGRP mAbs. The results showed that erenumab was effective despite the inclusion of patients with refractory cases. Considering the background of this study, which included refractory patients, we focused on the ≥30% MMD reduction rate and analyzed early and late responders. Previous real-world studies of erenumab over 3–6 months reported ≥30% response rates of 41–71% [[13], [14], [15]], comparable to those at 3–6 months in this study (47%–68%). However, in the 48-week study of erenumab by Barbanti et al. [12], the ≥50% response rate was 56% for patients with HFEM and 76% for patients with CM, which were superior to the rates observed in our study (20% at month 3 and 47% at month 6). In that study, 63% of patients chose to increase the dose from 70 mg to 140 mg. Erenumab is available in two doses, 70 mg and 140 mg per month, in the United States and Europe. The recommended starting dose of erenumab is 70 mg once per month, which can be increased to 140 mg once per month if the response is inadequate. In contrast, in Japan, the approved dose of erenumab is only 70 mg per month, which is based on the results of randomized controlled trials (RCTs) [10]. Clinical data suggest that erenumab 140 mg/month may be preferable to 70 mg/month in patients with EM or CM for whom previous prophylactic treatments have failed [21]. In addition, one female patient who was mistakenly given a dose of erenumab that exceeded the therapeutic dose (triple dose) had a more effective clinical response with no adverse effects [22]. Therefore, in this study, the use of the 70 mg erenumab dose approved in Japan may have influenced the efficacy of erenumab.

A single-center observational study of the effect of erenumab in 300 patients with CM found a sustained ≥30% response in 34% of patients for 52 weeks [23]. In a real-world clinical study of erenumab in 100 migraine patients, 24% had a sustained ≥30% response over 6 months, and 20% of those without an initial response had a delayed response in 3–6 months [24]. In our study, the average of ≥30% responders over 6 months was satisfactory, and 62.2% of all patients had no response in the first 3 months (average < 30%), of whom 46.4% had a response in the last 3 months (average ≥ 30%). Of note, among those switching from other CGRP mAbs, 74.1% had no response in the first 3 months (average < 30%), of whom 58.8% had a late response in the last 3 months (average ≥ 30%) (Fig. 5). We believe that our findings provide useful information in deciding when to continue erenumab in patients switching from other CGRP mAbs (fremanezumab or galcanezumab). In this study, the 1–3 months of observation of patients switching from other CGRP mAbs was not sufficient to determine efficacy, and 4–6 months of observation may be necessary. Additionally, the cumulative percentage of patients achieving a ≥30% response over 6 months ranged from 83 to 89% in the total, CGRP naïve, and CGRP mAb-switching groups (Fig. 4), indicating the importance of continuing treatment with erenumab. In a real-world study, half to two-thirds of patients converted from CM to EM after receiving erenumab [18,25]. In this study, the percentage of patients with CM decreased from 78% at the beginning of the study to 41% after 6 months, suggesting a beneficial effect of erenumab.

Overeem et al. found ≥30% response rates of 35% and 45% at 3 and 6 months, respectively, in 20 patients who were switched from a CGRP ligand mAb to erenumab [26]. In a multicenter study of 25 patients who were refractory to erenumab and switched to CGRP ligand mAbs (galcanezumab and fremanezumab), the ≥30% and ≥50% response rates were 32% and 12% at 3 months, respectively [27]. CGRP has been shown experimentally to bind not only to CGRP receptors but also to receptors for amylin and adrenomedullin. In contrast, adrenomedullin and amylin may activate the CGRP receptor [[28], [29], [30]]. Thus, the CGRP receptor mAb and the CGRP ligand mAb may block or stimulate several non-CGRP pathways to different degrees, producing individual differences in the response to CGRP ligand mAbs and CGRP receptor mAbs.

In our study, the presence of photophobia was an associated factor for responders throughout 1–6 months. In a real-world study of galcanezumab, a CGRP mAb, the presence of photophobia was reported as an associated factor for responders [31]. Light aversion behavior is induced by central and peripheral CGRP injections in wild-type mice and can be treated with triptans [32]. Therefore, sensory hypersensitivity symptoms, such as photosensitivity, may be an indicator of the efficacy of CGRP ligand or CGRP receptor mAbs.

Real-world studies tend to report a higher incidence of wearing-off effects than RCTs. This tendency may be because the intervals between visits are less strict in real-world studies than in RCTs, and comorbid diseases are more varied in real-world studies. A wearing-off effect was seen in 14–34% of patients in previous clinical studies of erenumab [33,34] but was seen to a lesser extent in this study (up to 12.5%). This is a favorable finding considering that the interval between doses of erenumab is not strictly defined in real-world clinical practice. Several real-world studies of erenumab have reported more adverse reactions than RCTs, including hypertension and gastrointestinal disorders [23,35,36]. In a study of 418 patients treated with erenumab, 51% had gastrointestinal problems, and one had a severe adverse event (small bowel obstruction) [36]. However, in this study, the safety profile was generally acceptable, with only 11% of patients experiencing adverse reactions, which were mild in severity. Four patients (8.9%) had constipation, but the degree was mild. Blood pressure was not strictly monitored, but no reports of elevated blood pressure were observed in our study. In addition, it is possible that minor adverse reactions were overlooked because of the retrospective design.

Study limitations include the relatively small sample size, single-center setting, and lack of efficacy measures other than MMD reduction. We did not assess the effect of erenumab on accompanying symptoms or the degree of disability caused by headache. Finally, sample size calculations were not performed for this study because this was an observational, retrospective study with an exploratory purpose. Our study results should be confirmed by well-designed larger studies of patients. Further studies are warranted to evaluate the frequency of drug administration and the role of different molecular targets for different mAbs.

## 8 Conclusion

Our study demonstrated the efficacy of erenumab in patients with migraine, including those switching from other CGRP mAbs and those with refractory migraine. At least 4–6 months may be preferable for efficacy evaluation in patients switching to erenumab from other CGRP mAbs.

## Ethics approval and consent to participate

The study was conducted in compliance with the Declaration of Helsinki and was approved by the Institutional Review Board of the Dokkyo Medical University Hospital (approval number: R-62-4J).

## Funding

The authors received no financial support for the research, authorship, and/or publication of this article.

## Data availability statement

The datasets from this study are available from the corresponding author upon reasonable request.

## CRediT authorship contribution statement

**Keisuke Suzuki:** Writing – original draft, Methodology, Formal analysis, Data curation, Conceptualization. **Shiho Suzuki:** Writing – review & editing, Methodology, Data curation, Conceptualization. **Tomohiko Shiina:** Writing – review & editing, Methodology, Data curation. **Yasuo Haruyama:** Writing – review & editing, Methodology, Formal analysis, Conceptualization. **Saro Kobayashi:** Writing – review & editing, Methodology, Conceptualization. **Mukuto Shioda:** Writing – review & editing, Methodology, Conceptualization. **Koichi Hirata:** Writing – review & editing, Supervision, Methodology, Data curation, Conceptualization.

## Declaration of competing interest

The authors declare the following financial interests/personal relationships which may be considered as potential competing interests: Keisuke Suzuki reports a relationship with Eli Lilly Japan, Daiichi Sankyo and Otsuka Pharmaceutical Co., Ltd. that includes: speaking and lecture fees. Shiho Suzuki reports a relationship with Eli Lilly Japan, Daiichi Sankyo, Amgen and Otsuka Pharmaceutical Co., Ltd that includes: speaking and lecture fees. Tomohiko Shiina reports a relationship with Eli Lilly Japan, Daiichi Sankyo, Amgen and Otsuka Pharmaceutical Co., Ltd that includes: speaking and lecture fees. Koichi Hirata reports a relationship with Eli Lilly Japan, Daiichi Sankyo, Amgen and Otsuka Pharmaceutical Co., Ltd that includes: speaking and lecture fees.
