# Supplementary material for: Real-world effectiveness of erenumab in Japanese patients with migraine
Source: Heliyon. 2024 Feb 17;10(4):e26568. doi: 10.1016/j.heliyon.2024.e26568 (PMC10900787; doi:10.1016/j.heliyon.2024.e26568)

Supplementary Figure 1: Mean changes in the number of monthly migraine days from baseline after erenumab treatment in 41 patients who completed 6 months of follow-up

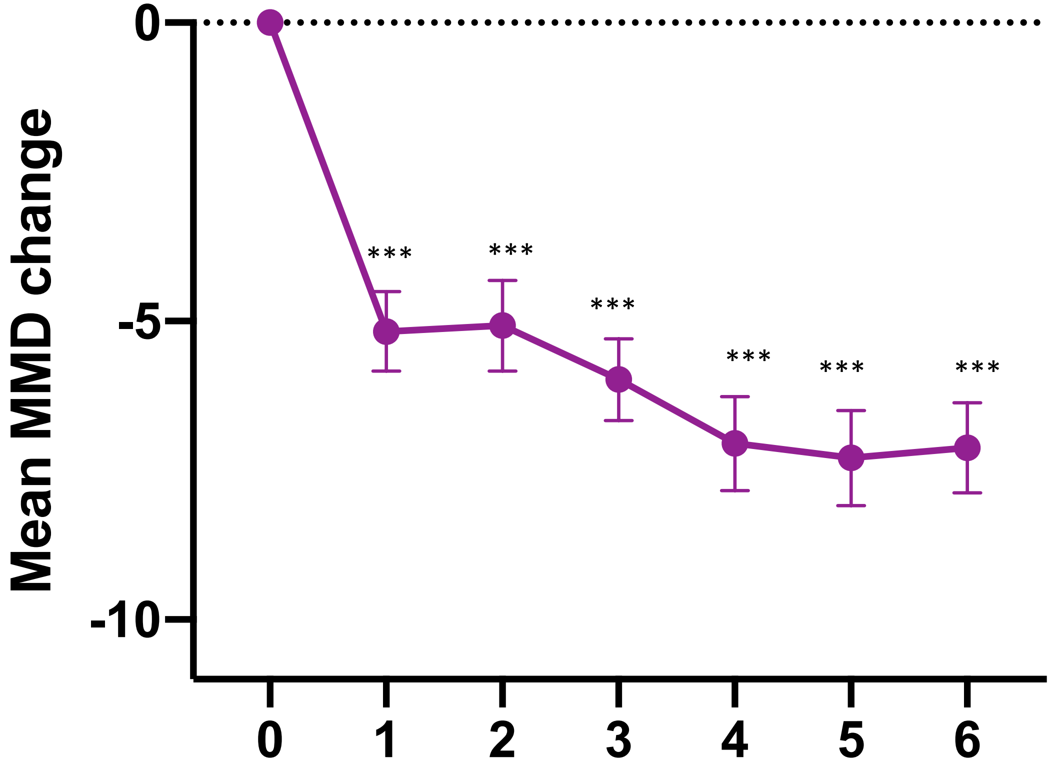

*** p<0.001, compared to baseline. Using a generalized linear mixed-effects model followed by Bonferroni's post hoc test

Supplementary Figure 2: Proportion of responders after erenumab treatment in 41 patients who completed 6 months of follow-up

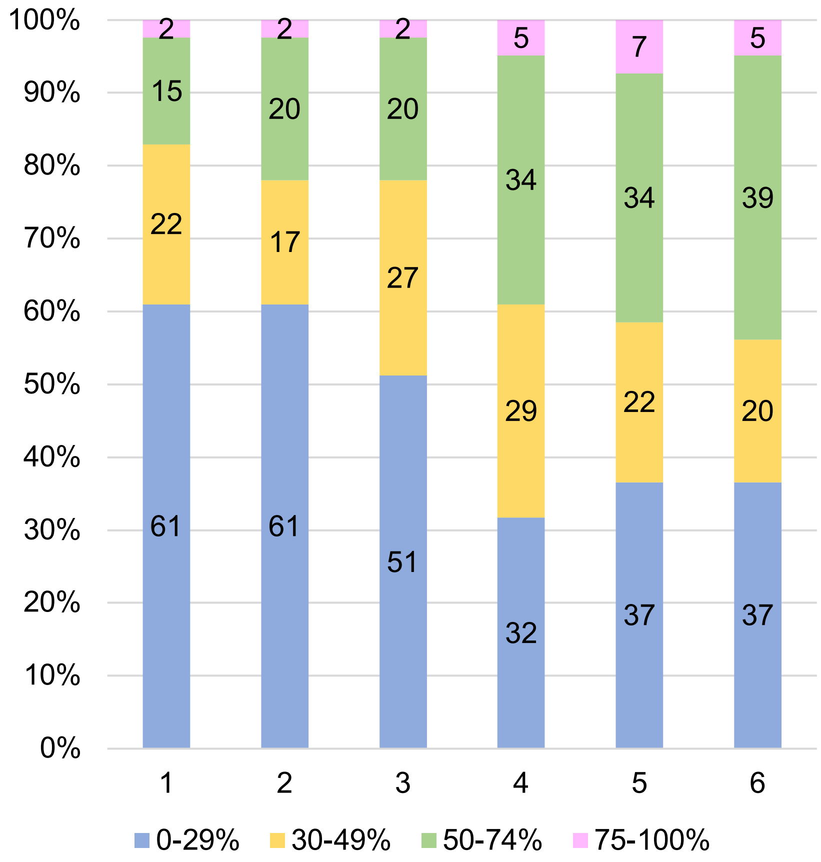

Supplement: Multimedia component 1 [file mmc1.pdf]
